# Indicators for Osteomyelitis in Children With Sickle Cell Disease Admitted With Vaso-Occlusive Crises

**DOI:** 10.7759/cureus.68265

**Published:** 2024-08-31

**Authors:** Duaa A Tashkandi, Ehab Hanafy, Norah Alotaibi, Doha Abuharfel, Ali Alnijaidi, Ahmed M Banjar, Fadwa Abufara, Shaima Riyad, Maisa Alhalabi, Naif Alblowi

**Affiliations:** 1 Pediatrics, King Salman Armed Forces Hospital, Tabuk, SAU; 2 Prince Sultan Oncology Center, King Salman Armed Forces Hospital, Tabuk, SAU; 3 Pediatric Medicine, King Salman Armed Forces Hospital, Tabuk, SAU; 4 Medicine, University of Tabuk, Tabuk, SAU; 5 Hematology and Oncology, King Salman Armed Forces Hospital, Tabuk, SAU; 6 Hematology and Oncology, Prince Sultan Oncology Center, King Salman Armed Forces Hospital, Tabuk, SAU

**Keywords:** osteo-articular infection, scd, sca, osteo-myelitis, voc

## Abstract

Introduction: Sickle cell disease (SCD) is an autosomal recessive genetic disorder characterized by the presence of a mutated form of hemoglobin (Hb) known as sickle hemoglobin (HbS). Individuals with SCD are susceptible to a variety of osteoarticular complications. Osteomyelitis is a commonly seen infection affecting the tibia, diaphysis of the femur and humerus, and vertebras.

Aim: The aim of this study was to define the indicators suggesting the diagnosis of osteomyelitis in patients with SCD.

Methods: This study is a descriptive, analytical, non-interventional, prospective study of pediatric patients with SCD admitted with vaso-occlusive crisis (VOC) and/or osteomyelitis, which were identified by laboratory and radiological features. Retrospective data was included for patients who met the inclusion criteria. The statistical analysis included a description of the primary and secondary outcomes in the cohort.

Results: A total of 28 children were included in this study. Participants' ages ranged from 11 months to 13 years. Males represented the majority (64.3%) of the participants. The blood culture of most of the participants (89.3%) showed no growth; however, 7.1% had salmonella, and only 3.6% had Gram-positive cocci. Most cases (75%) had leukocytosis. Thrombocytosis was present mainly in patients with VOC (40%). CRP was 1-4.9 mg/dL, mainly in patients with osteomyelitis (50%). The ferritin level exceeded 5000 ng/mL in patients with osteomyelitis or both osteomyelitis and VOC (50%). Ultrasound examinations revealed no hip effusion in 24 of the 28 examined patients. A plain X-ray examination showed no abnormality in 24 out of the 28 examined cases; with MRI, three cases exhibited marrow edema with bone enhancement, two (66.7%) were complicated by osteomyelitis, and the last (33.3%) had osteomyelitis and VOC. Aspiration was performed only in seven of the 28 examined, of which six (85.7%) were complicated by osteomyelitis, while the last one (14.3%) had acute chest syndrome.

Conclusion: Based on the outcomes of this study, we recommend an individualized multidisciplinary examination (hematology, infectious disease, orthopedic surgery, and interventional radiology) for SCD patients with suspected osteomyelitis admitted with VOC, considering the entire clinical history and laboratory and MRI results.

## Introduction

Sickle cell disease (SCD) is defined as an autosomal recessive hereditary condition characterized by the presence of sickle hemoglobin (HbS), a mutant type of hemoglobin (Hb). Individuals with SCD are susceptible to various osteoarticular complications [1-2]. These include dactylitis, vaso-occlusive crisis (VOC), osteomyelitis, avascular necrosis, and septic arthritis. In addition, due to the combination of ischemia and impaired immunity, the susceptibility to bacterial infections is high, causing osteomyelitis and septic arthritis. 

The acute painful crisis that occurs as early as six months of age and often lasts a lifetime is the predominant clinical hallmark of SCD. VOC is the most frequent reason for emergency department visits and hospitalizations [3].

Osteomyelitis is a frequent illness that affects the tibia, femoral and humeral diaphysis, and vertebrae. The gold standard tools to confirm the diagnosis include bone biopsy with histopathological examination and tissue culture [4]. Osteomyelitis is highly suspicious in patients with localized tenderness associated with high inflammatory markers and radiological findings. 

Salmonella is the most prevalent incidental pathogen in patients with SCD, followed by Staphylococcus aureus, which is found in 10% of patients with osteomyelitis [5]. A literature review by Burnett et al. showed that Salmonella is the most common cause of osteomyelitis in patients with SCD in developing and developed countries. This incidence is twice more than that of S aureus. Other bacterial species responsible for the infection include Enterobacter spp., Hemophilus influenza, and Escherichia coli [6].

Sickle cell anemia (SCA) is a homozygous type of HbS (HbSS) caused by a single-point substitution of glutamine by valine at the globin chain position [7]. In more than one-fourth of patients, acute pain is the first sign of the condition [8]. Bone involvement is the most common manifestation of SCA in an acute form, such as in VOC, osteomyelitis, or chronic progressive, such as in avascular necrosis [9].

A randomized controlled trial done by Berger et al. found significant predictors of osteomyelitis, which is an early presentation with fever alone without any pain or swelling in the affected limb. As a result, such individuals should be continuously observed and evaluated to rule out osteomyelitis [10].

A prospective study by Fontalis et al. suggests that for children with SCD presenting with persistent bone pain, fever, and elevated CRP and CBC, osteomyelitis should be suspected, and prompt antibiotic treatment should be started [11]. Therefore, this study aimed to define the indicators suggesting the diagnosis of osteomyelitis in patients with SCD.

Aim of the study

The purpose of this study was to define the indicators that can suggest the diagnosis of osteomyelitis in patients with SCD and investigate the laboratory and imaging pattern of osteomyelitis.

## Materials and methods

A prospective cohort study that included children with confirmed SCD by Hb electrophoresis was conducted in King Salman Armed Forces Hospital (KSAFH), a tertiary hospital covering the northwestern region of Saudi Arabia. Prospective data were included for patients who met the inclusion criteria. This study was conducted over six months after obtaining the required approval. 

The study’s inclusion criteria were patients with SCD confirmed by Hb electrophoresis, as well as patients experiencing a vaso-occlusive crisis (VOC) who were highly suspected of having osteomyelitis during their hospital course. Additionally, children with SCD who presented with localized tenderness, elevated inflammatory markers, and radiological findings indicative of osteomyelitis, as well as those with confirmed osteomyelitis through positive bone aspiration culture, were included in the study. Exclusion criteria included adult patients, defined as those over the age of 14 years, and patients admitted with manifestations other than VOC.

Data were collected from patients’ files, the hospital information system (HIS), and the picture archiving and communication system (PACS). IBM SPSS Statistics for Windows, Version 23, (Released 2015; IBM Corp., Armonk, New York, United States) was used for the statistical analysis. It included a description of the primary and secondary outcomes in the cohort. Descriptive statistics were also employed to characterize the baseline demographic information and clinical features. A p-value of <0.05 was considered statistically significant.

Ethical consideration

The study was approved by the academic affairs ethical committee at KSAFH, Tabuk, Saudi Arabia, with the approval number: KSAFH-REC-2021-425. Patient medical files and consent were not obtained, as no identifying information, including photos or imaging that could reveal their identity, was disclosed.

## Results

A total of 28 individuals met the inclusion criteria and were included in the study analysis. Although participants' ages ranged from 11 months to 13 years, the majority (39.3%) were older than 11 years. Male participants comprised the majority (64.3%), with females making up 35.7% (Table 1). Most participants' blood cultures (89.3%, N=25) showed no growth; however, 7.1% (N=two) tested positive for Salmonella, and 3.6% (N=one) had Gram-positive cocci (Table 2). Regarding leukocytic count, only 25% (N=seven) had a normal count, while 32.1% (N=nine) had 10,000 to 14,900 WBC/mm³, and 7.1% (N=two) exceeded 25,000 WBC/mm³. Furthermore, 60.7% (N=17) of patients presented with a platelet count of 150-450 x 10⁹/L, while 35.7% (N=10) had platelets exceeding 450 x 10⁹/L, and only one patient (3.6%) had a platelet count less than 150 x 10⁹/L. Measuring CRP in the participants showed that most patients (28.6%, N=eight) had ≤0.9 mg/dL, while 14.3% had 1-9.9 mg/dL, and 10.7% had CRP ≥20 mg/dL. The erythrocyte sedimentation rate (ESR) was elevated in most cases, being more than 60 mm/hr in 42.9% of cases (N=12). Most patients (50%, N=14) presented with ferritin levels of 7-140 ng/mL, while fibrinogen was 401-600 mg/dL in 53% (N=15) of cases. Albumin levels ranged between 34 g/L and 55 g/L, with the highest frequency (92.9%, N=26) being 41-50 g/L (Table 2).

**Table 1:** Demographic data This table represents the demographic data of the study participants. Data are presented as numbers and their corresponding percentages.

N=28		N	%
Age (years)	≤ 2	2	7.10%
	2- 5	4	14.30%
	> 5- 8	3	10.70%
	> 8- 11	8	28.60%
	> 11 years	11	39.30%
Gender	Male	18	64.30%
	Female	10	35.70%

**Table 2:** Blood culture and lab results This table represents the blood culture and laboratory results of the study participants. Data are presented as numbers and their corresponding percentages.

N= 28		N	%
Blood culture	No growth	25	89.30%
	Gram-positive methicillin-resistant staph epidermidis	1	3.60%
	Salmonella	2	7.10%
WBC in thousands/mm^3^	5 - 9.9	7	25.00%
	10-14.9	9	32.10%
	15-19.9	8	28.60%
	20-24.9	2	7.10%
	≥ 25	2	7.10%
Platelet count X 10^9^/L	< 150	1	3.60%
	150-450	17	60.70%
	> 450	10	35.70%
CRP (mg/dL)	≤ 0.9	8	28.60%
	1- 4.9	4	14.30%
	5- 9.9	4	14.30%
	10- 14.9	6	21.40%
	15- 19.9	3	10.70%
	≥ 20	3	10.70%
Erythrocyte sedimentation rate (ESR) (mm/hour)	≤ 10	5	17.90%
	11- 20	2	7.10%
	21- 40	4	14.30%
	41- 60	5	17.90%
	> 60	12	42.90%
Ferritin (ng/mL)	7-140	14	50.00%
	141- 300	3	10.70%
	301- 600	3	10.70%
	601- 1000	2	7.10%
	1001- 2000	3	10.70%
	2001- 5000	1	3.60%
	> 5000	2	7.10%
Fibrinogen (mg/dL)	< 200	4	14.30%
	200- 400	9	32.10%
	401- 600	15	53.60%
Albumin (g/L)	34-40	1	3.60%
	41-50	26	92.90%
	51-55	1	3.60%

Only 25% (N=seven) of the examined cases had a normal WBC count, whereas most cases exhibited leukocytosis. The count was 10-14.9 thousand WBCs/mm³, primarily in osteomyelitis cases (44%, N=four). It reached 15-19.9 thousand WBCs/mm³, mainly in patients with osteomyelitis or VOC complications (37.5%, N=three). A count of 20-24.9 thousand WBCs/mm³ occurred in patients without complications or with osteomyelitis and VOC (50%, N=one). The count exceeded 25 thousand WBCs/mm³ in patients with osteomyelitis or VOC complications (50%, N=one). Thrombocytopenia (platelet count <150 x 10⁹/L) was present in one patient with both osteomyelitis and VOC (100%, N=one). Thrombocytosis (platelet count >450 x 10⁹/L) was observed mainly in patients with VOC (40%, N=four), osteomyelitis (20%, N=two), and both osteomyelitis and VOC (10%, N=1). However, it also occurred in patients without complications (30%, N=three). Lab results showed normal CRP levels (≤0.9 mg/dL) in eight out of 28 patients. CRP levels of 1-4.9 mg/dL were mainly found in patients with osteomyelitis (50%, N=two) and both osteomyelitis and VOC, or hemolytic crisis (25%, N=one). CRP levels of 5-9.9 mg/dL were seen in patients without complications, with osteomyelitis, or with VOC (33.3%, N=one). CRP levels of 15-19.9 mg/dL were found in patients with osteomyelitis, VOC, or both (33.3%, N=one). CRP levels ≥25 mg/dL were observed in patients with osteomyelitis or VOC (50%, N=1).

Normal ESR levels (≤20 mm/hr) were found in only seven out of 28 examined patients. ESR levels of 21-40 mm/hr were observed in patients with osteomyelitis (25%, N=one), VOC (25%, N=one), and both osteomyelitis and VOC (50%, N=two). ESR levels of 41-60 mm/hour were seen in patients with osteomyelitis (60%, N=three) and in those with osteomyelitis or acute chest syndrome (20%, N=one). ESR levels exceeding 60 mm/hour were present in most examined cases, including patients without complications (16.7%, N=two), with osteomyelitis (50%, N=six), VOC (8.3%, N=one), and both osteomyelitis and VOC (25%, N=three).

Ferritin levels were normal (7-140 ng/mL) in most examined cases (14 out of 28 patients). However, levels exceeded 5000 ng/mL in patients with osteomyelitis or both osteomyelitis and VOC (50%, N=one).

A normal fibrinogen level (200-400 mg/dL) was found in nine out of 28 examined patients. It was observed in patients without complications or with osteomyelitis (33.3%, N=three), as well as in patients with VOC, osteomyelitis, and VOC, and patients with a hemolytic crisis (11.1%, N=one). A low fibrinogen level (<200 mg/dL) was found in four cases, including those without complications or with osteomyelitis (25%, N=one) and patients with VOC (50%, N=two). Most cases exhibited high fibrinogen levels (401-600 mg/dL) in 15 out of 28 examined cases, with the highest percentage in patients with osteomyelitis (40%, N=six).

Albumin levels in the examined cases were mostly in the high normal range (41-50 g/L) in 26 out of 28 cases (Table 3). An ultrasound examination revealed no hip effusion in 24 out of 28 examined cases. However, minimal hip effusion was present in cases without complication, patients with osteomyelitis and VOC, and patients with acute chest syndrome (33.3%, N=one). Pus collection was observed only in one case with osteomyelitis (100%, N=one).

**Table 3:** Correlation between lab results and complications This table represents the correlation between laboratory results and complications among the study participants. Data are presented as numbers and their corresponding percentages. A p-value is considered significant when it is less than 0.05. VOC: vaso-occlusive crisis; ESR: erythrocyte sedimentation rate

		No complication		Osteomyelitis		VOC		VOC + osteomyelitis		Hemolytic crisis		Acute chest syndrome		Total		p-value
		N	%	N	%	N	%	N	%	N	%	N	%	N	%	
WBC count (thousands / mm3)	5 - 9.9	0	0.0%	2	28.6%	2	28.6%	2	28.6%	1	14.3%	0	0.0%	7	100.0%	0.583
	10-14.9	3	33.3%	4	44.4%	0	0.0%	1	11.1%	0	0.0%	1	11.1%	9	100.0%	
	15-19.9	1	12.5%	3	37.5%	3	37.5%	0	0.0%	0	0.0%	1	12.5%	8	100.0%	
	20-24.9	1	50.0%	0	0.0%	0	0.0%	1	50.0%	0	0.0%	0	0.0%	2	100.0%	
	≥ 25	0	0.0%	1	50.0%	1	50.0%	0	0.0%	0	0.0%	0	0.0%	2	100.0%	
	Total	5	17.9%	10	35.7%	6	21.4%	4	14.3%	1	3.6%	2	7.1%	28	100.0%	
Platelet count (X 10^9^/L)	<150	0	0.0%	0	0.0%	0	0.0%	1	100.0%	0	0.0%	0	0.0%	1	100.0%	0.231
	150-450	2	11.8%	8	47.1%	2	11.8%	2	11.8%	1	5.9%	2	11.8%	17	100.0%	
	>450	3	30.0%	2	20.0%	4	40.0%	1	10.0%	0	0.0%	0	0.0%	10	100.0%	
	Total	5	17.9%	10	35.7%	6	21.4%	4	14.3%	1	3.6%	2	7.1%	28	100.0%	
CRP (mg/dL)	≤ 0.9	2	25.0%	2	25.0%	3	37.5%	0	0.0%	0	0.0%	1	12.5%	8	100.0%	0.674
	1- 4.9	0	0.0%	2	50.0%	0	0.0%	1	25.0%	1	25.0%	0	0.0%	4	100.0%	
	5- 9.9	1	33.3%	1	33.3%	1	33.3%	0	0.0%	0	0.0%	0	0.0%	3	100.0%	
	10- 14.9	2	33.3%	3	50.0%	0	0.0%	0	0.0%	0	0.0%	1	16.7%	6	100.0%	
	15- 19.9	0	0.0%	1	33.3%	1	33.3%	1	33.3%	0	0.0%	0	0.0%	3	100.0%	
	≥ 20	0	0.0%	1	33.3%	1	33.3%	1	33.3%	0	0.0%	0	0.0%	3	100.0%	
	Total	5	17.9%	10	35.7%	6	21.4%	4	14.3%	1	3.6%	2	7.1%	28	100.0%	
ESR (mm/hr)	≤ 10	2	40.0%	0	0.0%	1	20.0%	0	0.0%	1	20.0%	1	20.0%	5	100.0%	0.302
	11- 20 mm/hr	1	50.0%	0	0.0%	1	50.0%	0	0.0%	0	0.0%	0	0.0%	2	100.0%	
	21- 40	0	0.0%	1	25.0%	2	50.0%	1	25.0%	0	0.0%	0	0.0%	4	100.0%	
	41- 60	0	0.0%	3	60.0%	1	20.0%	0	0.0%	0	0.0%	1	20.0%	5	100.0%	
	> 60	2	16.7%	6	50.0%	1	8.3%	3	25.0%	0	0.0%	0	0.0%	12	100.0%	
	Total	5	17.9%	10	35.7%	6	21.4%	4	14.3%	1	3.6%	2	7.1%	28	100.0%	
Ferritin (ng/mL)	7-140	2	14.3%	5	35.7%	4	28.6%	2	14.3%	0	0.0%	1	7.1%	14	100.0%	0.095
	141- 300	2	66.7%	1	33.3%	0	0.0%	0	0.0%	0	0.0%	0	0.0%	3	100.0%	
	301- 600	1	33.3%	0	0.0%	0	0.0%	1	33.3%	1	33.3%	0	0.0%	3	100.0%	
	601- 1000	0	0.0%	2	100.0%	0	0.0%	0	0.0%	0	0.0%	0	0.0%	2	100.0%	
	1001- 2000	0	0.0%	1	33.3%	2	66.7%	0	0.0%	0	0.0%	0	0.0%	3	100.0%	
	2001- 5000	0	0.0%	0	0.0%	0	0.0%	0	0.0%	0	0.0%	1	100.0%	1	100.0%	
	> 5000	0	0.0%	1	50.0%	0	0.0%	1	50.0%	0	0.0%	0	0.0%	2	100.0%	
	Total	5	17.9%	10	35.7%	6	21.4%	4	14.3%	1	3.6%	2	7.1%	28	100.0%	
Fibrinogen (mg/Dl)	< 200	1	25.0%	1	25.0%	2	50.0%	0	0.0%	0	0.0%	0	0.0%	4	100.0%	0.495
	200- 400	3	33.3%	3	33.3%	1	11.1%	1	11.1%	1	11.1%	0	0.0%	9	100.0%	
	401- 600	1	6.7%	6	40.0%	3	20.0%	3	20.0%	0	0.0%	2	13.3%	15	100.0%	
	Total	5	17.9%	10	35.7%	6	21.4%	4	14.3%	1	3.6%	2	7.1%	28	100.0%	
Albumin (g/L)	34-40	0	0.0%	0	0.0%	0	0.0%	1	100.0%	0	0.0%	0	0.0%	1	100.0%	0.363
	41-50	4	15.4%	10	38.5%	6	23.1%	3	11.5%	1	3.8%	2	7.7%	26	100.0%	
	51-55	1	100.0%	0	0.0%	0	0.0%	0	0.0%	0	0.0%	0	0.0%	1	100.0%	
	Total	5	17.9%	10	35.7%	6	21.4%	4	14.3%	1	3.6%	2	7.1%	28	100.0%	

A plain X-ray examination showed no abnormality in 24 out of 28 examined cases. Periosteal reactions were present in three cases with osteomyelitis complications. Soft tissue swelling was encountered in one case with osteomyelitis. A normal lumbar lordotic curve loss was observed in one case with osteomyelitis and VOC. Additionally, repeated white lung was found in one case with VOC.

An MRI examination revealed no abnormality in 24 out of 28 examined cases. However, three cases exhibited marrow edema with bone enhancement, two (66.7%, N=two) had osteomyelitis and one (33.3%, N=one) had both osteomyelitis and VOC. Three cases showed soft tissue edema, two (66.7%, N=two) had osteomyelitis and one (33.3%, N=1) had both osteomyelitis and VOC. Three cases presented with joint effusion and soft tissue edema, all with osteomyelitis complications. Two cases showed soft tissue edema and subperiosteal collection, one with osteomyelitis and the other with VOC. Muscle edema appeared in MRI examinations of two cases, one with osteomyelitis and the other with both osteomyelitis and VOC. Joint effusion was present in two MRI findings, one with osteomyelitis and VOC and the other with acute chest syndrome. The subperiosteal collection was present in one case with osteomyelitis (Table 4).

**Table 4:** Correlation between radiological findings, surgical intervention, and complications This table represents the correlation between radiological findings, surgical interventions, and complications among the study participants. Data are presented as numbers and their corresponding percentages. A p-value is considered significant when it is less than 0.05. VOC: vaso-occlusive crisis

		No complication		Osteomyelitis		VOC		VOC+ Osteomyelitis		Hemolytic crisis		Acute chest syndrome		Total		P value
		N	%	N	%	N	%	N	%	N	%	N	%	N	%	
Ultrasound	No hip effusion	4	16.7%	9	37.5%	6	25.0%	3	12.5%	1	4.2%	1	4.2%	24	100.0%	0.607
	Minimal hip effusion	1	33.3%	0	0.0%	0	0.0%	1	33.3%	0	0.0%	1	33.3%	3	100.0%	
	Pus collection	0	0.0%	1	100.0%	0	0.0%	0	0.0%	0	0.0%	0	0.0%	1	100.0%	
	Total	5	17.9%	10	35.7%	6	21.4%	4	14.3%	1	3.6%	2	7.1%	28	100.0%	
X-ray	No abnormality	5	22.7%	6	27.3%	5	22.7%	3	13.6%	1	4.5%	2	9.1%	22	100.0%	0.601
	Soft tissue swelling	0	0.0%	1	100.0%	0	0.0%	0	0.0%	0	0.0%	0	0.0%	1	100.0%	
	Periosteal reaction	0	0.0%	3	100.0%	0	0.0%	0	0.0%	0	0.0%	0	0.0%	3	100.0%	
	Loss of normal lumbar lordotic curve	0	0.0%	0	0.0%	0	0.0%	1	100.0%	0	0.0%	0	0.0%	1	100.0%	
	Repeated white lung	0	0.0%	0	0.0%	1	100.0%	0	0.0%	0	0.0%	0	0.0%	1	100.0%	
	Total	5	17.9%	10	35.7%	6	21.4%	4	14.3%	1	3.6%	2	7.1%	28	100.0%	
MRI	No abnormality	5	41.7%	0	0.0%	5	41.7%	0	0.0%	1	8.3%	1	8.3%	12	100.0%	0.281
	Marrow edema with bone enhancement	0	0.0%	2	66.7%	0	0.0%	1	33.3%	0	0.0%	0	0.0%	3	100.0%	
	Subperiosteal collection	0	0.0%	1	100.0%	0	0.0%	0	0.0%	0	0.0%	0	0.0%	1	100.0%	
	Soft tissue edema	0	0.0%	2	66.7%	0	0.0%	1	33.3%	0	0.0%	0	0.0%	3	100.0%	
	Soft tissue edema + subperiosteal collection	0	0.0%	1	50.0%	1	50.0%	0	0.0%	0	0.0%	0	0.0%	2	100.0%	
	Muscle edema	0	0.0%	1	50.0%	0	0.0%	1	50.0%	0	0.0%	0	0.0%	2	100.0%	
	Joint effusion + soft tissue edema	0	0.0%	3	100.0%	0	0.0%	0	0.0%	0	0.0%	0	0.0%	3	100.0%	
	Joint effusion	0	0.0%	0	0.0%	0	0.0%	1	50.0%	0	0.0%	1	50.0%	2	100.0%	
	Total	5	17.9%	10	35.7%	6	21.4%	4	14.3%	1	3.6%	2	7.1%	28	100.0%	
Surgical intervention	No	5	23.8%	4	19.0%	6	28.6%	4	19.0%	1	4.8%	1	4.8%	21	100.0%	0.028
	Aspiration	0	0.0%	6	85.7%	0	0.0%	0	0.0%	0	0.0%	1	14.3%	7	100.0%	
	Total	5	17.9%	10	35.7%	6	21.4%	4	14.3%	1	3.6%	2	7.1%	28	100.0%	

Aspiration was performed in seven out of 28 examined cases; six of these (85.7%, N=six) had osteomyelitis complications, while one (14.3%, N=one) had acute chest syndrome (Table 4).

## Discussion

SCD is a genetic condition characterized by defective, sickle-shaped erythrocytes [12] Because of their structure, these erythrocytes are more likely to become caught in small, slow-flowing capillaries, resulting in vaso-occlusive disease. Since this usually occurs in the bones, patients with SCD are more likely to have orthopedic symptoms such as osteomyelitis, septic joint, or osteonecrosis. Osteomyelitis is a dangerous and possibly crippling illness, yet it can be difficult to distinguish it from milder SCD disorders like VOC [13]. An osteomyelitis diagnosis necessitates a comprehensive examination of the clinical presentation, laboratory tests, and imaging. The treatment of osteomyelitis in SCD patients can be medical or surgical, but antibiotic selection and care must be considered preoperatively and postoperatively to promote the best outcomes. To our knowledge, few studies reviewed cases of osteomyelitis in pediatric patients with SCD and have used a case-control method to compare the presentation of osteomyelitis with that of VOC, which is the most common cause of bony pain in these children. Other studies have involved adult patients, and their findings may not apply to children [14,15]. In this study, we aimed to define the indicators that can suggest the diagnosis of osteomyelitis in patients with SCA and investigate the laboratory and imaging pattern of osteomyelitis.

In this study, the blood culture of most of the participants (89.3%) showed no growth; however, 7.1% had salmonella and only 3.6% had Gram-positive cocci. Bloodstream infections (BSIs) are more common in children with SCD, particularly with encapsulated bacteria such as Streptococcus pneumoniae and Hemophilus influenza. As with osteomyelitis, this infection risk is associated with decreased or missing splenic function, other immunological abnormalities, and anatomic predisposition [16,17]. The Cooperative Study of SCD, conducted before routine penicillin prophylaxis or pneumococcal immunizations, provided a prospective longitudinal assessment of bacteremia risk, revealing that incidence rates were highest in patients with HbSS aged three years, owing primarily to S pneumoniae and H influenzae, and they decreased with age during the first two decades of life [18]. Later investigations, from 1993 to 2010, revealed S pneumoniae, Salmonella, E. coli, and Staphylococcus aureus as sources of bacteremia in SCD [19].

Concerning leucocytic count, only 25% had an average count, 32.1% had 10000 to 14900 WBC/mm3, and 7.1% exceeded 25000/ mm3. Awogu et al. [20] reported that when compared to their M genotype controls, steady-state SCA children had substantially higher mean total white blood cell counts, mean total polymorphonuclear neutrophil counts, and mean total segmented and mean total non-segmented neutrophil counts. Contrary to expectations, steady-state patients showed a low percentage of polymorphonuclear neutrophils (34%) that did not alter substantially with age.

Only 25% of the examined cases had normal WBC counts. However, most cases had leukocytosis. Thrombocytopenia (platelet count < 150 X 109/L) was present in one patient with osteomyelitis and VOC. However, thrombocytosis (platelet count >450 X 109/L) was present in patients, mainly in patients with VOC (40%), with osteomyelitis (20%), and in patients with both osteomyelitis and VOC (10%). 

CRP lab results were normal ≤ 0.9 mg/dL only in eight out of 28 patients. However, it was 1-4.9 mg/dL, mainly in patients with osteomyelitis (50%) and patients with osteomyelitis and VOC, or patients with hemolytic crisis (25%). Sickle cell illness is an inflammatory disorder characterized by unusually high leukocyte counts [21] and higher levels of WBCs during and after VOC or acute chest syndrome [22]. Recent data suggest that chronic inflammation plays a role in SCA development [23]. Increased levels of pro-inflammatory markers, such as high-sensitivity C-reactive protein (hs-CRP), were seen in the sera of SCA patients [24]. Circulating CRP levels are typically steady, regulated by the synthesis rate, and within a typical range for each individual. The identification of elevated hs-CRP levels in the sera of steady-state SCA patients, which increased significantly during VOC episodes, showed a correlation between CRP and VOC [22].

Normal ESR level ≤20 mm/hour was found in only seven of the 28 patients examined. The ESR was 41-60 mm/hour in patients with osteomyelitis (60%) and those with osteomyelitis or acute chest syndrome (20%). In a retrospective analysis by Weisman et al. [25], it was shown that at presentation, 143% of the 28 patients treated with osteomyelitis had leukocytosis (>15 000/L), and 46% had significantly increased baseline CRP (>10 mg/dL). Baseline ESR was raised (>20 mm/h) in 27 patients (96%), although only three patients (11%) had severe ESR elevation (>100 mm/h). Baseline white blood cell counts in patients treated for osteomyelitis were not substantially different from those in individuals not treated for osteomyelitis but were lower in patients with probable/confirmed osteomyelitis. Baseline CRP was considerably higher in patients treated for osteomyelitis compared to patients not treated for it but not statistically different in patients with probable/confirmed osteomyelitis compared to patients not treated for osteomyelitis. On the other hand, ESR was considerably greater in patients treated for osteomyelitis compared to individuals not treated for osteomyelitis. This held even when the comparison was confined to patients with probable/confirmed osteomyelitis [23].

The ferritin level was normal (7-140 ng/mL) in most examined cases. However, it exceeded 5000 ng/mL in patients with osteomyelitis or both osteomyelitis and VOC (50%). Other studies [24] found that individuals with SCA had higher levels of serum iron than the control group. Iron is a strong oxidant capable of converting hydrogen peroxide to free radicals. When these free radicals are created, they have the potential to cause oxidative damage to cells by damaging lipids, proteins, and DNA. As a result, greater iron content is harmful to cells and causes substantial oxidative damage. Higher blood iron levels in HbSS VOC patients may be due to the fast auto-oxidation of the HbS molecule as well as the severe intravascular hemolysis observed in such patients. As a result, it is probable that oxidative stress is greater in patients with HbSS VOC than in those with HbSC VOC [26].

An ultrasound (US) examination revealed no hip effusion in 24 of the 28 examined. However, minimal hip effusion was present in cases without complication, patients with osteomyelitis and VOC, and patients with acute chest syndrome (33.3%, N=one). Pus collection was observed only in one case complicated by osteomyelitis (100%, N=one). US is highly sensitive in detecting osteomyelitis in children with SCD [14,24]. In a study by Inusa et al. [27], the US sensitivity was 76%, with patients having a 24% false negative rate, which was later validated with a repeat US/MRI in cases of elevated white blood cell count and/or CRP [28]. According to William et al. [29], the US result of 4 mm sub-periosteal fluid accumulation significantly predicts osteomyelitis in SCD patients. In our osteomyelitis group, 32% of patients exhibited periosteal elevation or fluid collection of more than 4 mm [30].

A plain X-ray examination showed no abnormality in 24 out of the 28 examined cases. However, periosteal reaction presented in three cases only complicated by osteomyelitis. Soft tissue swelling was encountered in only one case complicated by osteomyelitis. Normal lumbar lordotic curve loss was observed in one case complicated by osteomyelitis and VOC. Moreover, repeated white lung was found in only one case complicated by VOC. Plain radiographs in the early stages of osteomyelitis and VOC are frequently unremarkable or only indicate soft-tissue edema, periostitis, or osteopenia [3,13]. On radiographs, the lytic alterations characteristic of osteomyelitis are at least two weeks behind the infection phase [31]. As a result, the low sensitivity and specificity of plain radiography for early identification necessitate further investigation using additional imaging modalities [30].

The examination with MRI revealed no abnormality in 24 out of the 28 examined cases. However, three cases exhibited marrow edema with bone enhancement; two (66,7%) were complicated by osteomyelitis, and the last (33.3%) had osteomyelitis and VOC. Three cases showed soft tissue edema; two (66,7%) were complicated by osteomyelitis, and the last (33.3%) had osteomyelitis and VOC. Three cases present with joint effusion and soft tissue edema. All cases are complicated by osteomyelitis. Two cases showed soft tissue edema and subperiosteal collection. One of them was complicated by osteomyelitis, and the other with VOC. Muscle edema appeared in the MRI examination of two cases, one was complicated by osteomyelitis and the other with both osteomyelitis and VOC. Joint effusion was present in two MRI findings, one was complicated by osteomyelitis VOC and the other with acute chest syndrome. A subperiosteal collection was present in only one case, which was complicated by osteomyelitis. Because of its high sensitivity, MRI is the imaging modality of choice for diagnosing osteomyelitis [25,29]. Early pathological signs, such as bone marrow edema, can be detected within 24 hours after infection [26]. Bone marrow edema on MRI often appears as a localized marrow anomaly with a reduced signal on T1-weighted scans and an increased signal on T2-weighted imaging [31]. Other secondary osteomyelitis appearances, such as soft-tissue collections, cellulitis, and cortical bone sinus tracts, exhibit comparable MRI signaling properties (lower on T1, higher on T2) [25,27,29]. MRIs can be used with clinical evidence to evaluate the response to antibiotic medication without the danger of ionizing radiation [32-34].

Aspiration was performed only in seven of the 28 examined cases; six (85.7%) were complicated by osteomyelitis, while the last one (14.3%) had acute chest syndrome. Given the limitations of diagnostic imaging techniques like bone scintigraphy, detecting osteomyelitis in children with SCD can be particularly challenging since they frequently appear with fever and a painful, swollen, sensitive limb with a limited range of motion. These signs and symptoms are comparable to those seen in VOC patients. However, a bone biopsy or aspiration is an intrusive treatment, so it should be conducted before commencing antibiotics to maximize the odds of achieving a positive result [10].

Study limitation

This study has several limitations that must be acknowledged. Firstly, the sample size was relatively small, which may affect the generalizability of the findings. The study was conducted in a single center, which could introduce location-specific biases and limit the applicability of the results to other settings. Additionally, the retrospective nature of part of the data collection may introduce recall bias and affect the accuracy of the findings. The reliance on clinical and radiological criteria for diagnosing osteomyelitis, without the routine use of gold-standard diagnostic methods such as bone biopsy, could lead to misclassification.

## Conclusions

Despite recent breakthroughs in diagnostic examinations, our findings suggest that the diagnosis of osteomyelitis in children with SCD remains challenging to establish with certainty. Misdiagnosis has significant implications for providers caring for children with SCD because undertreatment can result in chronic osteomyelitis, necessitating prolonged antibiotic therapy, and surgical interventions. In contrast, overtreatment can result in unnecessary exposure to broad-spectrum antibiotics and prolonged hospitalizations. Based on the outcomes of this study and prior research, we recommend an individualized multidisciplinary examination (hematology, infectious disease, orthopedic surgery, and interventional radiology) in SCD patients with suspected osteomyelitis, considering the entire clinical history and laboratory and MRI results.

## References

[1] Serjeant GR (2013). The natural history of sickle cell disease. Cold Spring Harb Perspect Med.

[2] Ejindu VC, Hine AL, Mashayekhi M, Shorvon PJ, Misra RR (2007). Musculoskeletal manifestations of sickle cell disease. Radiographics.

[3] Almeida A, Roberts I (2005). Bone involvement in sickle cell disease. Br J Haematol.

[4] Fritz JM, McDonald JR (2008). Osteomyelitis: approach to diagnosis and treatment. Phys Sportsmed.

[5] Sinkin JC, Wood BC, Sauerhammer TM, Boyajian MJ, Rogers GF, Oh AK (2015). Salmonella osteomyelitis of the hand in an infant with sickle cell disease. Plast Reconstr Surg Glob Open.

[6] Burnett MW, Bass JW, Cook BA (1998). Etiology of osteomyelitis complicating sickle cell disease. Pediatrics.

[7] Al Farii H, Zhou S, Albers A (2020). Management of osteomyelitis in sickle cell disease: review article. J Am Acad Orthop Surg Glob Res Rev.

[8] Fassihi SC, Lee R, Quan T, Tran AA, Stake SN, Unger AS (2020). Total hip arthroplasty in patients with sickle cell disease: a comprehensive systematic review. J Arthroplasty.

[9] Bainbridge R, Higgs DR, Maude GH, Serjeant GR (1985). Clinical presentation of homozygous sickle cell disease. J Pediatr.

[10] Berger E, Saunders N, Wang L, Friedman JN (2009). Sickle cell disease in children: differentiating osteomyelitis from vaso-occlusive crisis. Arch Pediatr Adolesc Med.

[11] Fontalis A, Hughes K, Nguyen MP, Williamson M, Yeo A, Lui D, Gelfer Y (2019). The challenge of differentiating vaso-occlusive crises from osteomyelitis in children with sickle cell disease and bone pain: a 15-year retrospective review. J Child Orthop.

[12] Ware RE, de Montalembert M, Tshilolo L, Abboud MR (2017). Sickle cell disease. Lancet.

[13] Vanderhave KL, Perkins CA, Scannell B, Brighton BK (2018). Orthopaedic manifestations of sickle cell disease. J Am Acad Orthop Surg.

[14] Sadat-Ali M (1998). The status of acute osteomyelitis in sickle cell disease. A 15-year review. Int Surg.

[15] Ebong WW (1986). Acute osteomyelitis in Nigerians with sickle cell disease. Ann Rheum Dis.

[16] Battersby AJ, Knox-Macaulay HH, Carrol ED (2010). Susceptibility to invasive bacterial infections in children with sickle cell disease. Pediatr Blood Cancer.

[17] Booth C, Inusa B, Obaro SK (2010). Infection in sickle cell disease: a review. Int J Infect Dis.

[18] Gill FM, Sleeper LA, Weiner SJ (1995). Clinical events in the first decade in a cohort of infants with sickle cell disease. Cooperative study of sickle cell disease. Blood.

[19] Baskin MN, Goh XL, Heeney MM, Harper MB (2013). Bacteremia risk and outpatient management of febrile patients with sickle cell disease. Pediatrics.

[20] Awogu AU (2000). Leucocyte counts in children with sickle cell anaemia usefulness of stable state values during infections. West Afr J Med.

[21] Chies JA, Nardi NB (2001). Sickle cell disease: a chronic inflammatory condition. Med Hypotheses.

[22] Gonçalves MS, Queiroz IL, Cardoso SA (2001). Interleukin 8 as a vaso-occlusive marker in Brazilian patients with sickle cell disease. Braz J Med Biol Res.

[23] Lanaro C, Franco-Penteado CF, Albuqueque DM, Saad ST, Conran N, Costa FF (2009). Altered levels of cytokines and inflammatory mediators in plasma and leukocytes of sickle cell anemia patients and effects of hydroxyurea therapy. J Leukoc Biol.

[24] Canellas CG, Carvalho SM, Anjos MJ, Lopes RT (2012). Determination of Cu/Zn and Fe in human serum of patients with sickle cell anemia using radiation synchrotron. Appl Radiat Isot.

[25] Weisman JK, Nickel RS, Darbari DS, Hanisch BR, Diab YA (2020). Characteristics and outcomes of osteomyelitis in children with sickle cell disease: a 10-year single-center experience. Pediatr Blood Cancer.

[26] Voskou S, Aslan M, Fanis P, Phylactides M, Kleanthous M (2015). Oxidative stress in β-thalassaemia and sickle cell disease. Redox Biol.

[27] Inusa BP, Oyewo A, Brokke F, Santhikumaran G, Jogeesvaran KH (2013). Dilemma in differentiating between acute osteomyelitis and bone infarction in children with sickle cell disease: the role of ultrasound. PLoS One.

[28] Krishnan S, Setty Y, Betal SG, Vijender V, Rao K, Dampier C, Stuart M (2010). Increased levels of the inflammatory biomarker C-reactive protein at baseline are associated with childhood sickle cell vasocclusive crises. Br J Haematol.

[29] William RR, Hussein SS, Jeans WD, Wali YA, Lamki ZA (2000). A prospective study of soft-tissue ultrasonography in sickle cell disease patients with suspected osteomyelitis. Clin Radiol.

[30] Martínez VB, González-Juanatey JR (2009). Markers of inflammation and cardiovascular disease: clinical applications of C-reactive protein determination. Am J Cardiovasc Drugs.

[31] Akohoue SA, Shankar S, Milne GL, Morrow J, Chen KY, Ajayi WU, Buchowski MS (2007). Energy expenditure, inflammation, and oxidative stress in steady-state adolescents with sickle cell anemia. Pediatr Res.

[32] Calhoun JH, Manring MM (2005). Adult osteomyelitis. Infect Dis Clin North Am.

[33] Lee YJ, Sadigh S, Mankad K, Kapse N, Rajeswaran G (2016). The imaging of osteomyelitis. Quant Imaging Med Surg.

[34] Pineda C, Espinosa R, Pena A (2009). Radiographic imaging in osteomyelitis: the role of plain radiography, computed tomography, ultrasonography, magnetic resonance imaging, and scintigraphy. Semin Plast Surg.

